# Sustainable selection of waste collection trucks considering feasible future scenarios by applying the stratified best and worst method

**DOI:** 10.1016/j.heliyon.2023.e15481

**Published:** 2023-04-14

**Authors:** Héctor Moreno-Solaz, Miguel-Ángel Artacho-Ramírez, Pablo Aragonés-Beltrán, Víctor-Andrés Cloquell-Ballester

**Affiliations:** Project Management, Innovation and Sustainability Research Center (PRINS), Universitat Politècnica de València, 46022 Valencia, Spain

**Keywords:** Municipal solid waste, Waste collection trucks, MCDM, Stratified best and worst method, Stratification, Sustainable mobility

## Abstract

Municipal solid waste (MSW) management is vital in achieving sustainable development goals. It is a complex activity embracing collection, transport, recycling, and disposal; and whose management depends on proper strategic decision-making. The use of decision support methods such as multi-criteria decision-making (MCDM) is widespread in MSW management. However, their application mainly focuses on selecting plant locations and the best technologies for waste treatment. Despite the critical role played by transport in promoting sustainability, MCDM has seldom been applied for the selection of sustainable transport alternatives in the field of MSW management. There are a few MCDM studies about choosing waste collection vehicles, but none that include the most recent green vehicles among the options or consider feasible future scenarios. In this article, different engine technologies for collection trucks (diesel, compressed natural gas (CNG), hybrid CNG-electric, electric, and hydrogen) are evaluated under sustainability criteria in a Spanish city by applying the stratified best and worst method (SBWM). This method enables considering the uncertainty associated with future events to establish various feasible scenarios. The results show that the best-valued options are electric and diesel trucks, in that order, followed by CNG and hybrid CNG-electric, and with hydrogen-powered trucks coming last. The SBWM has proven helpful in defining a comprehensive framework for selecting the most suitable engine technology to support long-term MSW collection. Considering sustainability among the criteria and feasible future scenarios in waste management collection decision-making provides more comprehensive and conclusive results that help managers and policymakers make better informed and more reliable decisions.

## 1 Introduction

All human activities should care for and preserve the environment by meeting current ecological requirements and standards. Studies and efforts to control the environmental impact of human actions have grown exponentially in recent years [1]. Hence, sustainability is gradually being consolidated as a premise in decision-making processes, especially in those expected to have a long-term impact. Municipal solid waste (MSW) management, defined as the management of domestic and commercial garbage generated under the jurisdiction of a municipal body [2] is not an exception. Waste disposal and waste handling are global problems [3] and many countries are implementing mitigation strategies in MSW to reduce greenhouse gas (GHG) emissions [4]. Additionally, MSW management is called to play a relevant role in the circular economy because landfills as end-of-life product receivers could promote remanufacturing and zero-waste implementations. As a result, sustainable waste management is crucial for reaching sustainable development goals and a more sustainable society [5]. For this reason, the collection, transfer, and transport of MSW are some of the most challenging tasks for local municipalities and represent a significant portion of municipal expenditure [6]. To pursue sustainability, MSW management must face complex mobility challenges. A well-designed transport system improves delivery operations and quality of life by reducing costs, resources, and energy consumption [7]. The collection of MSW is carried out by varyingly sized collecting vehicles working within schedules that collect waste from bins in the streets until full. The waste is then transported to treatment plants or intermediate transfer stations using powerful trucks that consume a lot of energy. This energy currently comes mainly from petroleum derivatives, specifically diesel, that produce high levels of GHG emissions and noise [8]. In addition, in recent years, European regulations on selective collection have forced municipalities to increase the number of routes and vehicles to collect five different fractions of waste (glass, paper & cardboard, packaging, biowaste, and mixed waste) [9,10]. Additionally, door-to-door collection is becoming more frequent in many municipalities since this system achieves the best separation ratios [11]. Therefore, the optimal design of routes, the location of transfer plants, and the type of collection truck are vital to obtaining cost-effective and environmentally friendly solutions [12]. However, although current technological alternatives for transport appear to be clean and sustainable, there is much uncertainty about their global ecological fit and long-term deployment [13]. Thus, apart from the interaction of economic, institutional, social, political, and environmental factors, there is uncertainty about regulation, normalization, and deployment of existing green vehicles. These factors and uncertainties make it difficult to find the suitable alternative that best fits real-world conditions.

In this scenario, multi-criteria decision-making (MCDM) methods are appropriate for choosing the best engine technology because they can concurrently synthesize these conflicting criteria and achieve a trade-off among them [14]. MCDA concepts, methods, and applications have been widely studied in the operational research literature [15,16]. Among the better-known models are those based on multiple attribute utility theory (MAUT) [17], analytic hierarchy process (AHP) [18] and analytic network process (ANP) [19], as well as outranking methods such as ELECTRE [20] or PROMETHEE [21] or a technique for order preference by similarity to ideal solution (TOPSIS) [22]. These methods assume that the decision-maker has a certain level of knowledge about the alternatives and the consequences of the choice. When there is vagueness or imprecision in the data or in the judgments in the context of the decision problem, fuzzy MCDM can be used [23].

MCDM has been extensively used for decades in various waste management problems and circumstances. However, its application mainly focuses on selecting suitable locations and waste treatment, disposal, and recycling technologies [24]. To the authors' knowledge, the selection of truck engine technologies, including the most recent green vehicles, has not yet been addressed. Moreover, the most applied MCDM methods in waste management have been AHP (47%), followed by ANP (9%), and VIKOR (7%) [24]. Studies including uncertainty management are less frequent (37%), and only one percent deal with the probability of different scenarios occurring [24]. After making a decision, the decision-maker frequently becomes hesitant about whether the proper weightings were assigned to the criteria, given that various possibilities may occur in the near future [25]. Thus, as a complex system operating in volatile, uncertain, complex, and ambiguous (VUCA) environments, waste management should develop plans to manage uncertainty considering feasible future scenarios.

This paper aims to select the best engine technology for waste collection trucks in the city of Castellon, considering sustainability and feasible future events that could in the long term affect the decision to be made at present. The paper applies a recent MCDM method, the stratified MCDM (SMCDM) [25], which is based on the best and worst method (BWM) [26] and enables computing weightings for criteria for feasible future scenarios. The starting hypothesis is that considering sustainability among criteria and feasible future scenarios in waste management collection decision-making will provide more comprehensive and conclusive results to help managers and policymakers make more informed and reliable decisions.

The remainder of this paper is structured as follows. Section 2 provides a background of previous academic works on MCDM methodologies in the context of waste collection and the research gap of the paper. In Section 3, the methodology of this research is presented, and Section 4 contains the main results of the application of this methodology in the city of Castellon (Spain) for selecting among vehicle motor options. A discussion of the results is then presented in Section 5. Finally, Section 6 provides the conclusions, the limitations of the study, and suggests research work.

## 2 Background

Multi-criteria decision-making (MCDM) involves a broad range of methods to support decision-making to reach a compromise when there are multiple criteria. The most used methodologies are the analytic hierarchy process (AHP), outranking procedures, and a technique for ordering preferences by similarity to an ideal solution (TOPSIS) [27].

Several studies use MCDM techniques to deal with waste management problems. Many of these studies focus on either locating waste treatment plants [[28], [29], [30], [31]] or on technological decisions within the broad field of waste treatment [14,[32], [33], [34]]. However, most only describe one scenario without considering the impact of future situations and the probabilities of occurrence.

A feasible future scenario is a possible or trending situation that can be imagined or achieved by applying different variables (including political, economic, social, and cultural). In this sense, SMCDM was created to consider feasible future scenarios in the decision-making process.

SMCDM is based on the concept of stratification (CST) introduced by Zadeh [35]. The author considered a series of stratum or multiple levels through which the system transits from the input to a given target state or desired level. The concept has proven helpful in logistic informatics [36], artificial intelligence, natural language processing, big data, and robotics [37]. Asadabadi [25] was the first author to apply the CST in MCDM, and first coined the term SMCDM in the literature by considering future events that might influence decision-making. Single and integrated uses of SMCDM can be found in the literature. It has been applied for humanitarian aid distribution center selection in a post-disaster planning phase [38], for long-term planning in flood risk management [39], for implementation of Industry 4.0 in the mobility sector [40], and for selecting sustainable circular suppliers [41].

Because of its newness, little research on applying SMCDM can be found for waste management. As stated by Torkayesh et al. [24], only one percent of the existing studies with uncertainty-based MCDM methods in this field use the concept of stratification. As remarkable exceptions, we can find the work of Torkayesh et al. [42], applying SMCDM for waste disposal technology selection, Torkayesh & Simic [43], using the method for recycling facility location and the work of Tirkolaee et al. [44], employing SMCDM for sustainable healthcare landfill location selection. However, a developing trend of using uncertainty-based MCDM methods has arisen [24] because their greater reliability and accuracy ensure more scientifically robust and informed decision-making processes. Specifically, SMCDM enables decision-makers to develop plans considering different scenarios, and uncertainty can be managed to select the option that best fits real-world conditions.

Regarding the choice of vehicles, several MCDM studies compare options for motorizing people transport [45,46]; or more recently, mobility sharing systems [47]. Furthermore, in the last few years, some authors have focused on aspects related to electric vehicles, such as multiple fuel supply systems [48], smart charging scheduling at workplaces [49], or the management of lithium-ion batteries at the end of their lives [50]. However, there is little academic literature on analyzing available technologies for waste collection truck motors using MCDM, and none that considers feasible future scenarios simultaneously.

Table 1 summarizes the literature review on the application of MCDM techniques for the collection and transport of waste. A survey has been carried out on the literature using the following keywords: MCDM; multi-criteria; waste collection; MSW; vehicles; trucks; technologies; fuel; electric; and transport.

**Table 1:** Application of MCDM techniques in waste collection and transportation.

Work	Country	Problem description	Simple MCDM	Hybrid MCDM	Assessed Alternatives	Considering future scenarios
[51]	USA	Rank fuel alternatives for waste collection vehicles	–	TOPSIS, SAW	Waste collection trucks	No
[52]	Turkey	Rank waste collection systems in a smart city	TOPSIS	–	Technologies for smart collection	No
[53]	Tunisia	Route planning with GIS tools	ELECTRE III	–	Route optimization	No
[54]	Serbia	Rank fuels for waste collection	WASPAS	–	Waste collection trucks	No
[55]	Egypt	Optimizing construction and demolition waste transportation	COPRAS OCRA	–	Number and volume of vehicles	No
[56]	India	Evaluate different collection alternatives	Linear optimization model	–	Cost-benefit vs home segregation degree	Yes
[57]	Saudi Arabia	Choose recycling collection method for recovered fiber	–	BWM, TOPSIS	MCDM results comparation	No
[58]	Pakistan	Provide a facilitating framework incorporating circular economy principles	–	SWARA, VIKOR	Critical facilitators for the adoption of smart waste management	No
[59]	Saudi Arabia	Complete solid waste collection system selection	T-SHFS	–	Smart technologies for waste collection	No
[60]	Turkey	Select most appropriate policy for small household appliance collection methods		–	Small household appliance collection systems	No
[61]	Spain	Design methodology to evaluate circularity alternatives for construct and demolition waste	VIKOR	–	Types of recycled concrete, influenced by transport distances	No
[62]	Canada	Design waste collection program	CBA	–	Levels of satisfaction	No
[63]	Bosnia and Herzegovina	Selecting the best municipal solid waste collection scenario	–	AHP, VIKOR	Degrees of waste separation	No
[64]	Iran	Assess environmental problems derived from petroleum products in transportation	PROMETHEE	–	Fuels for light-duty vehicles	Yes
[65]	China	Locate a recyclable waste transportation vehicle parking center	–	DEMATEL, EW, WASPAS	Facility location	No
[66]	Poland	Planning of waste management systems in urban areas	–	Selection compromise programming	Waste management systems and waste fractions	Yes
[67]	Turkey	Analysis of location selection Problem for underground waste containers	–	MAIRCA, MABAC	Waste container location	No
[68]	Iran	Design transportation system in industrial waste management	–	BWM, PROMETHEE	Routes and fleet optimization	No
[69]	Iran	Define new model for urban waste collection and energy generation	–	NSGA-II, MOPSO	Integrated waste management models	No
[70]	Iran	Minimize the transportation cost and maximize the suitability	–	DELPHI, TOPSIS, E-CONSTRAINT	Holistic decision support tool	No
[71]	Malaysia	Define a Route optimization method combinate with GIS	AHP	–	Route optimization	No
[72]	Turkey	Evaluate smart waste collection systems based on internet of things	–	CODAS and IVq-ROFSs	Smart waste collection alternatives	No
[73]	Italy	Minimize operational costs and environmental impact of MSW management (heuristic approaches)	BWM	–	Algorithms for routes optimization	No
[74]	India	Mitigate the interacting barriers to online e-waste collection platforms	DEMATEL	–	Strategies for mitigating existing barriers	No
[75]	Italy	Evaluate smart reverse logistics development scenarios	–	Fuzzy DANP and fuzzy COBRA	Scenarios integrating Industry 4.0 technologies	No
[76]	India	Evaluate different collection alternatives	AHP	–	MSW collection and transportation methods and vehicles	No

As can be seen in Table 1, none of these academic works apply techniques that consider the occurrence of feasible future scenarios using the stratified BWM (SBWM). It is possible using CST to assess each criterion under each foreseen scenario [25]. This approach provides this research with considerable potential because it enables important decisions to be made, such as selecting waste collection trucks by considering feasible future events that will probably affect the final suitability of choice.

### 2.1 Research gap and contributions

The inclusion of uncertainty management in MCDM applied to waste management is still far from frequent – but the number of examples continue to grow given its reliable results. Using the concept of stratification (CST) and considering future scenarios in the decision-making process is even less frequent and practically residual in MSW [24]. To the authors’ knowledge, no MCDM study has included the most recent green vehicles as alternatives for selecting truck engine technologies in MSW management. Thus, the present work tries to fill these gaps in the literature by applying the SMCDM to choose the most suitable engine technology for MSW collection trucks in the city of Castellon. Moreover, with the inclusion of sustainability criteria in the decision-making process, the paper defines a comprehensive framework to select the most suitable engine technology for long-term MSW collection.

## 3 Methodology

The objective is to select the best engine technology for waste collection trucks considering feasible future events. A MCDM known as the stratified best and worst method (SBWM) has been chosen [42] as it considers feasible future scenarios when prioritizing available alternatives. The SBWM is an extension of the best and worst method (BWM), which is used to assess and compare the criteria chosen in each possible scenario.

### 3.1 The stratified best and worst method (SBWM)

The best and worst method is a popular multi-criteria decision-making (MCDM) method developed by Rezaei [26] which solves the inconsistency problem generated with AHP [77]. Torkayesh et al. [42] recently extended this method, considering different future scenarios, and developed the SBWB as follows:

Step 1 Determine a set of decision criteria $\left\{c_{1},c_{2},..,c_{n}\right\}$ that should be used to arrive at a decision.

For example, when choosing a house, the criteria could be C1, size; C2, availability of public transportation; and C3, price.

Step 2 Possible future scenarios are identified because they can change the decision-making process.

Following the example, three scenarios could be S1, current situation; S2, family growth; and S3, workplace change.

Step 3 Probabilities for transitioning between scenarios are assessed to build the transition probability matrix. In other words, experts determine the likelihood of the occurrence of each scenario based on historical data and their expertise.

Simplifying the probabilities could be 50% in the first case, 30% in the second, and 20% in the third. The probability matrix would be (0.5, 0.3, 0.2).

Step 4 Based on expert knowledge, determine the best (e.g., most desirable, most important) and worst (e.g., least desirable, least important) criteria for each scenario. No comparison is made at this stage.

For example, the best criteria in the first scenario could be price, and the worst could be size. This evaluation must be made in each scenario because each can differ.

Step 5 Determine, for each scenario, the preference of the best criterion over other criteria using a number between 1 and 9, according to this scale:

1: equal importance, 2: between equal and moderate, 3: moderately more important than, 4: between moderate and strong, 5: strongly more important than, 6: between strong and very strong, 7: very strongly more important than, 8: between very strong and absolute, 9: absolutely more important than.

The resulting best-to-others vector would be $A_{B}=\left(a_{B1,}a_{B2,}\ldots ,a_{Bn}\right)$, where $a_{Bj}$_j_ indicates the preference of the best criterion B over criterion j. It is clear that $a_{BB}=1$.

Making the pairwise comparison, an example of a best-to-others vector for the first scenario could be (7,3,1). The same is done in each of the three scenarios.

Step 6 Determine, for each scenario, the preference of all the criteria over the worst criterion using a number between 1 and 9, with the scale of step 5. The resulting others-to-worst vector would be ${A_{W}=\left(a_{1W,}a_{2W,}\ldots ,a_{nW}\right)}^{T}$, where $a_{jW}$ indicates the preference of the criterion j over the worst criterion w, and $a_{WW}=1$.

In the example, the others-to-worst vector in the first scenario could be (1,4,6). The same is done in each of the three scenarios.

Step 7 Find the optimal weights for each scenario ${(w}_{1}^{*},w_{2}^{*},\ldots ,w_{n}^{*})$. The optimal weight for the criteria is the one where, for each pair of $w_{B}/w_{j}$ and $w_{j}/w_{W}$, we have $w_{B}/w_{j}=a_{Bj}$ and $w_{j}/w_{W}=a_{jW}$. To satisfy these conditions for all j, we should find a solution where the maximum absolute differences $\left|\frac{w_{B}}{w_{j}}-a_{Bj}\right|$ and $\left|\frac{w_{j}}{w_{W}}-a_{jW}\right|$ for all j is minimized. Considering the non-negativity and sum condition for the weights, Eq. (1) results:

$$\min\begin{array}{c} \max\\ j \end{array}\left\{\left|\frac{w_{B}}{w_{j}}-a_{Bj}\right|,\left|\frac{w_{j}}{w_{W}}-a_{jW}\right|\right\}\text{,}$$

s.t.

(1) $$\sum_{j}w_{j}=1$$

$w_{j}\geq 0$, for all j.

This can be transferred to the Eq. (2):

min ξ.

s.t.

(2) $$\left|\frac{w_{B}}{w_{j}}-a_{Bj}\right|\leq \xi ,for\;all\;j$$

$\left|\frac{w_{j}}{w_{W}}-a_{jW}\right|\leq \xi$*,* for all j.

$$\sum_{j}w_{j}=1$$

$w_{j}\geq 0$, for all j.

Optimal weights ${(w}_{1}^{*},w_{2}^{*},\ldots ,w_{n}^{*})$ and $\xi^{*}$ are then obtained.

Following with the example,

$$\left|\frac{w_{3}}{w_{1}}-7\right|\leq \xi \text{,}$$

$$\left|\frac{w_{3}}{w_{2}}-3\right|\leq \xi \text{,}$$

$$\left|\frac{w_{2}}{w_{1}}-4\right|\leq \xi \text{,}$$

$$w_{1}+w_{2}+w_{3}=1\text{,}$$

$$w_{1},w_{2},w_{3}\geq 0\text{.}$$

So, the weights in the first scenario would be.

$$w_{1}=0.0909,w_{2}=0.2545,w_{3}=0.6545,\xi =0.4010$$

The same procedure must be followed in each scenario to build a matrix criteria/scenario (see Table 2).

**Table 2:** Example of weights of criteria in each scenario.

Criteria	S1	S2	S3
C1	0.0909	0.0600	0.3260
C2	0.2545	0.2560	0.1890
C3	0.6545	0.6840	0.4850

Step 8 The consistency ratio "*CR*" provides a measure of the consistency of a comparison. This ratio is calculated in each scenario using (3).

(3) $$Consistency\;ratio\;\left(CR\right)=\frac{\xi }{Consistency\;index}$$

In the example, for the consistency ratio, $a_{Bw}=a_{31}=7$, the consistency index is 3.37 [26] so the CR=0.4010/3.37=0.1190, which means good consistency. The same procedure must be repeated in each scenario.

Step 9 Multiply the weightings in the scenarios using the transition probability matrix to obtain the optimal weights of the criteria.

Once we have the weights in each scenario, we build the matrix (Table 2).

We then multiply the matrix by the transition probability matrix (see step 3), resulting in the optimal criteria weights: C1, 0.1287; C2, 0.2419 and C3, 0.6295.

Step 10 Construct an alternative criteria matrix again using SBWM. In this matrix, each option is evaluated with respect to the selection criteria, and a score is computed for each. Applying BWM in each alternative, an example of an alternative normalized decision matrix with two alternatives (house "A" and house "B") is shown in Table 3.

**Table 3:** Example of the alternative normalized decision matrix.

Criteria	House "A"	House "B"
C1	0.4280	0.5720
C2	0.3680	0.6320
C3	0.5040	0.4960

Step 11 Multiply the alternative normalized decision matrix by the optimal weights of the criteria matrix. These will be the final values showing the preference for each technology.

Finally, in the example proposed, we multiply the Table 3 matrix by the optimal criteria weights. By doing this, we obtain the final values of our alternatives: house "A" (0.4613) and house "B" (0.5386).

### 3.2 Research methodology

Once the chosen method is explained, the methodology phases created for this work are:

**Phase I.** Description of the problem and the specific case study of the city of Castellon.

**Phase II.** Selection and description of the decision support experts.

**Phase III.** Identification of the technologies used in waste collection trucks and the method of choosing them, identifying the main available technologies to be evaluated (the alternatives).

**Phase IV.** Selection of the criteria for choosing a technology based on an academic review and decision-maker experience.

**Phase V.** Analysis of the feasible future scenarios that will play a role when prioritizing the alternatives.

**Phase VI.** Definition of the probability occurrence of these feasible scenarios and assessing each criterion by applying BWM (SBWM).

**Phase VII.** Determination of the optimal weights for each criterion, multiplying the weights obtained in each scenario by the probability of their occurrence.

**Phase VIII.** BWM application for assessing each of the evaluated technologies (alternatives) for each criterion and answering the question: which technology is better for each of the chosen criteria?

**Phase IX.** Ranking the available technologies from highest to lowest according to the scores obtained by multiplying the matrices obtained in phases VII and VIII.

**Phase X.** Conducting a sensitivity analysis.

**Fig. 1:**
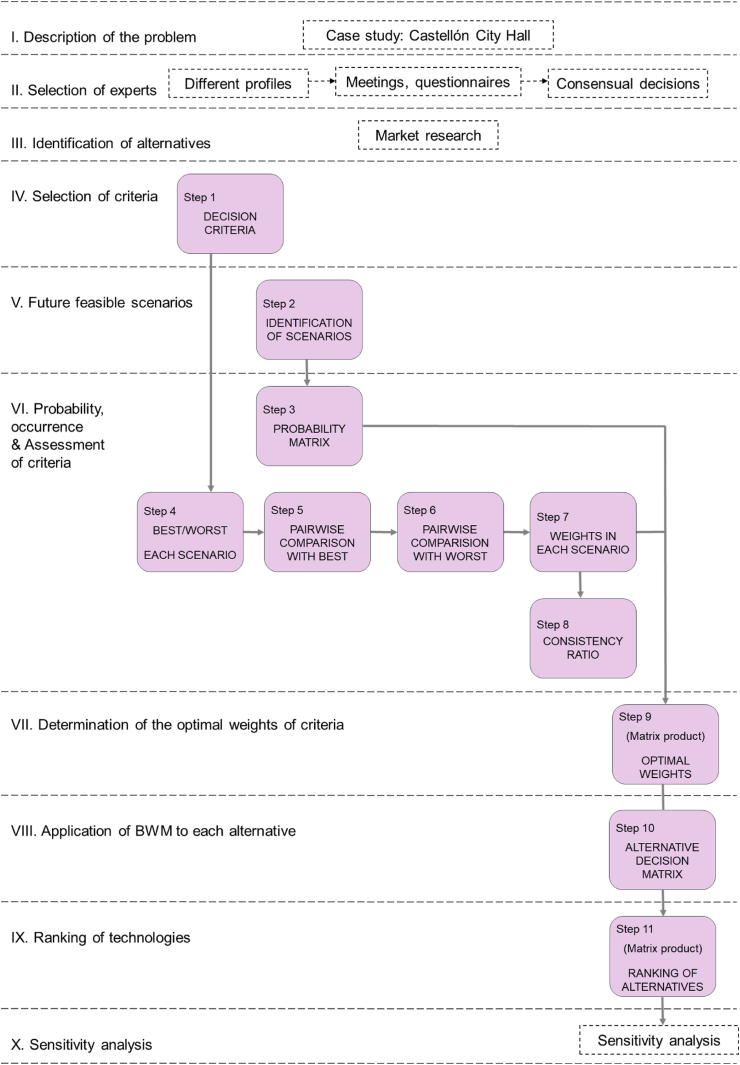
Phases of the methodology.

Fig. 1 shows a graphical scheme of the methodology followed.

## 4 Case study: Castellon City Council, Spain

Castellon is a Spanish Mediterranean city (**Phase I**). It is the capital of the province of Castellon and is in the north of the Valencia Region with a population of 172,589 [78]. Waste generation in the city exceeds 405 kg per inhabitant and year [79]. Collection is divided into five fractions according to current regulations [80] with more than 6900 containers – a containerization ratio of 74.7 L per inhabitant. Most collection trucks are diesel or CNG and have been in use for over ten years. For this reason, Castellon City Council is considering replacing the fleet.

A group of experts was created (**Phase II**). This group was constituted of five senior managers, one from each of the five main MSW management companies in Eastern Spain, three municipal engineers, two PhD engineers from the Jaime I University, two PhD engineers from Universitat Politècnica de València, a representative from the local transport association, an executive of the Valencian Energy Association, two local political representatives, and four environmental educators from the provincial MSW management board. It is also important to point out that all the scores introduced in the steps of the multi-criteria method – and the decisions made – were established consensually.

First, an informative meeting was held with the experts to gather the necessary data. The main objective was to obtain an initial proposal of technological alternatives, criteria, and scenarios for the case study. The facilitator then wrote and explained the proposal and sent it by email to the experts. Once this part was confirmed, in a second meeting, a questionnaire was delivered to establish the probability of occurrence of each of the scenarios and the criteria were compared in each. In the questionnaire, the questions for each scenario were phrased in this style:

In your opinion, what is the probability of occurrence for the different events? Which are the most/least important criteria in this scenario? Finally, compare for each scenario and using the following scale, all the rest of the criteria with the best and worst criterion.

In a third meeting, a similar questionnaire was delivered. This time the objective was to establish the expert opinions regarding the chosen technologies, and so the following question was asked for each of the criteria:

In your opinion, which is the best/worst technology for C1? Finally, compare in each criterion, using the following scale, all the other technologies with the best and the worst technology.

The experts then identified several technologies available for truck engines and evaluated the options. Currently, the leading technologies available (**Phase III**) in Europe for garbage trucks are diesel (T1), compressed natural gas – CNG (T2), hybrid electric-CNG (T3), electric (T4), and hydrogen (T5).

A series of relevant criteria (**Phase IV**) were then defined as extracted from the scientific literature and confirmed by expert experience. These criteria, fundamental when selecting an engine technology, are the following:

C1: cost of buying the vehicle [51].

C2: operating cost, that is, the servicing cost of the truck (insurance, tires, fuel, etc.) [81].

C3: polluting atmospheric emissions [82,83].

C4: truck noise [84].

C5: social acceptance of the technology and its use [42].

C6: availability of spare parts [54].

C7: estimated lifetime of the truck. Based on their experience, the group of experts determined that this criterion was crucial for making this kind of decision.

C8 is the ease of refueling (number of service stations, refueling times, etc.) [51,54].

C9 is flexibility in the vehicle's configuration (such as the possibility of mounting several axles, boxes, reduced chassis, and bicompartments) [85].

**Fig. 2:**
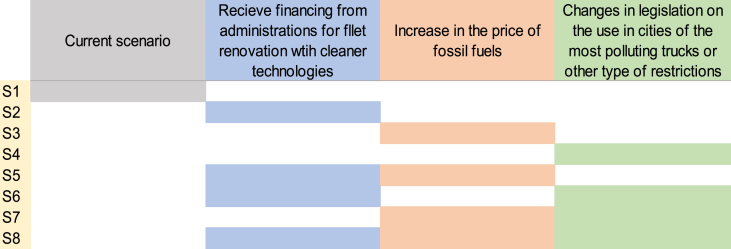
Definition of scenarios.

When setting the feasible scenarios, the choice is not limited to the current scenario and so eight scenarios are considered (**Phase V**) to determine the choice of truck technology by considering the probability of each occurring. To determine these future scenarios, the experts considered those foreseen events with a high probability of occurrence that could have meaningful repercussions on the decision process. To do this, they created a list of trends by analyzing the sector and market situation, current technologies, and legislation. They then observed the evolution of other sectors that have impacted or could directly or indirectly impact on waste management. When determining future expectations, the mentioned eight scenarios were established – from the most conservative scenario to a scenario that presents significant changes. Finally, the time horizon set by the experts was ten years as this is usually the amortization period for machinery in waste management services [86]. The eight scenarios obtained are shown in Fig. 2.

The occurrence probabilities for the scenarios were estimated by the experts consensually and based on their experience and observations of trends in recent years (**Phase VI**). Probabilities of 10%, 60%, 75%, and 80% were established for scenarios S1, S2, S3 and S4, respectively. The scenario with the smallest probability value (10%) is called "pS1". The remaining scenario probabilities are expressed as a function of "pS1". Considering that scenarios are independent situations, the probability of scenarios S5, S6, S7, and S8 is obtained as a product of their probabilities.

Finally, considering that the sum of the probabilities of the contemplated scenarios must be equal to 1, the following equation can be created:

360pS1^3^+153pS1^2^+22,5pS1 = 1

where pS1 = 0.03528.

Table 4 shows the transition probability between the defined scenarios based on this data.

**Table 4:** Transition probability of scenarios.

Scenario	pS1	pS2	pS3	pS4	pS5	pS6	pS7	pS8
Transition probability	0.0353	0.2117	0.2646	0.2822	0.0560	0.0597	0.0747	0.0158

As shown in Table 4, the experts have determined that the most foreseeable scenario for the next ten years is a series of regulatory changes in the field of waste management (S4). They have also allocated a high probability for increased fossil fuel prices (S3).

Once the transition probability is calculated, the experts determine consensually the score each criterion should have for each scenario (**Phase VI**). The objective in this phase is for the decision-maker to complete Table 5, choosing the best and worst criteria for each scenario and then comparing the rest of the criteria with both in pairs.

**Table 5:** SBWM. Scores of each scenario.

States	S1	S2	S3	S4	S5	S6	S7	S8
Best criterion	C1	C2	C2	C3	C2	C3	C3	C3
Worst criterion	C5	C5	C5	C5	C5	C5	C5	C5
**Best to others**								
C1	1	4	3	1	4	4	2	4
C2	2	1	1	1	1	2	2	2
C3	3	2	2	1	2	1	1	1
C4	3	2	2	2	2	3	3	3
C5	4	3	4	4	3	4	4	4
C6	3	2	3	2	2	2	2	2
C7	3	3	2	2	2	2	2	2
C8	2	2	2	2	2	2	2	2
C9	3	3	3	3	3	3	3	3
**Others to the worst**								
C1	8	3	8	7	4	4	8	4
C2	8	8	9	7	9	8	8	9
C3	6	7	4	9	6	9	9	9
C4	4	5	3	6	5	5	4	4
C5	1	1	1	1	1	1	1	1
C6	5	6	5	6	6	7	7	7
C7	5	6	6	6	6	6	6	7
C8	4	5	6	5	5	6	6	6
C9	5	5	4	4	4	4	4	3

After applying the BWM solver, the weights obtained for each scenario are shown in Table 6. The consistency index "*CR*" is close to zero for all of them, which means that the obtained results are robust.

**Table 6:** Weights of criteria based on SBWM.

Criteria	S1	S2	S3	S4	S5	S6	S7	S8
C1	0.2019	0.0677	0.0889	0.1440	0.0647	0.0673	0.1261	0.0678
C2	0.1442	0.1805	0.1778	0.1920	0.1727	0.1345	0.1261	0.1355
C3	0.0962	0.1353	0.1333	0.1920	0.1295	0.1883	0.1765	0.1848
C4	0.0962	0.1353	0.1333	0.0960	0.1295	0.0897	0.0840	0.0903
C5	0.0288	0.0301	0.0222	0.0240	0.0288	0.0269	0.0252	0.0246
C6	0.0962	0.1353	0.0889	0.0960	0.1295	0.1345	0.1261	0.1355
C7	0.0962	0.0902	0.1333	0.0960	0.1295	0.1345	0.1261	0.1355
C8	0.1442	0.1353	0.1333	0.0960	0.1295	0.1345	0.1261	0.1355
C9	0.0962	0.0902	0.0889	0.0640	0.0863	0.0897	0.0840	0.0903
*CR*	0.0865	0.0902	0.0889	0.0480	0.0863	0.0807	0.0756	0.0862

In the S1 current scenario (no change), investment costs achieve the highest weight for the decision-maker. However, in scenarios S2, S3, S4, and S5, operating costs obtain a higher weight in the decision-making process. In the rest of the scenario combinations (S6, S7, and S8), the criterion with the highest weight is atmospheric emissions (C3).

The matrix product in Table 4, Table 6 is then calculated to obtain the optimal weights for each criterion (**Phase VII**). The result is shown in Table 7.

**Table 7:** Optimal weights of criteria.

Criteria	C1	C2	C3	C4	C5	C6	C7	C8	C9
Weight	0.1037	0.1738	0.1561	0.1147	0.0255	0.1095	0.1117	0.1230	0.0820

Considering all the scenarios and their transition probabilities, it can be generally observed that the highest optimal weight is obtained by the operating cost criterion (C2).

The next step (**Phase VIII**) establishes which technology (T1, T2, T3, T4, and T5) is best for each criterion. For this, the technical characteristics provided by the truck manufacturers were analyzed, and BWM was again applied to obtain the alternative normalized decision matrix (Table 8).

**Table 8:** Alternative normalized decision matrix.

Criteria	T1. Diesel	T2. CNG	T.3 CNG-Electric	T4. Electric	T5. Hydrogen
C1	0.4213	0.2528	0.1011	0.1685	0.0562
C2	0.0800	0.1600	0.2400	0.4000	0.1200
C3	0.0396	0.0808	0.1347	0.3407	0.4041
C4	0.0406	0.1168	0.2335	0.3756	0.2335
C5	0.0374	0.0935	0.2336	0.4019	0.2336
C6	0.3043	0.2174	0.2174	0.2174	0.0435
C7	0.2677	0.3780	0.1890	0.0394	0.1260
C8	0.4757	0.1822	0.1822	0.1093	0.0506
C9	0.5000	0.2055	0.1233	0.1233	0.0479

For the decision-maker, the diesel truck is the most appropriate in four of the nine criteria (C1, C6, C8, and C9), and it stands out in terms of flexibility and ease of refueling. The electric truck obtains the best weights in three categories (C3, C4, and C5) and stands out in social acceptance.

**Fig. 3:**
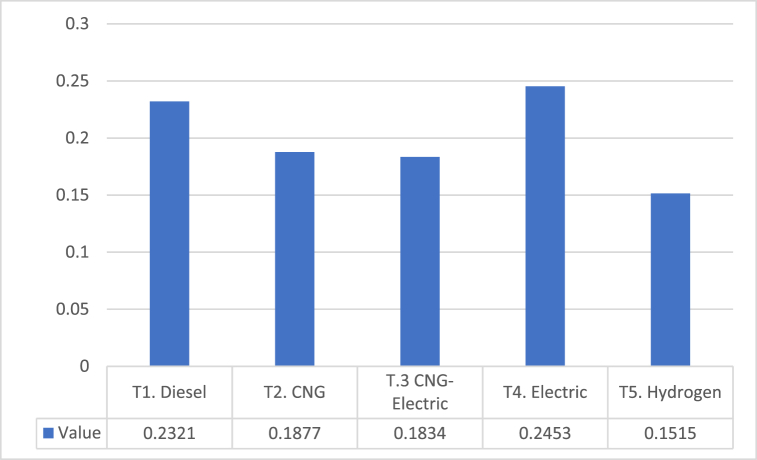
Ranking of alternatives.

Finally (**Phase IX**), to choose the best technology, the optimal weights of each criterion (Table 7) are multiplied by the alternative normalized decision matrix (Table 8). Fig. 3 shows the ranking of the alternatives.

In light of the results, where the differences between the first alternative and the second are small, a sensitivity analysis is proposed (**Phase X**). The process followed consists of developing an algorithm where the weight of a given criterion is successively modified in several steps, keeping the sum of criteria equal to 1. What is added or subtracted from the weight in each step of the reference criterion, is added or removed to the other criteria according to the initial proportion. At each step, the ranking of alternatives is recalculated, allowing us to observe how the orders are modified when modifying the weight of the reference criterion.

The results show a similar trend for criteria C1, C8, and C9 (see supplementary material). When the weight of these criteria is increased slightly from their original values, the T4 (electric) quickly falls in position, while the T1 (diesel) gains very quickly. In the case of criterion C6, this tendency is also fulfilled, but it is less pronounced than for the previous criteria.

The opposite happens with criteria C2, C4, and C5. When their weight is increased from the original values, technology T4 (electric) increases its ranking quickly while T1 (diesel) decreases.

In the case of C3, the more its weight increases from the original, the more the ranking of T4 (electric) increases. But simultaneously, the ranking of T5 (CNG-electric) increases, and when the weight of C3 reaches approximately 0.65, the alternative T5 takes first place. T1 (diesel) falls to last position in this situation.

In the case of C7, when its weight increases from its original value, the ranking of T4 (electric) drops rapidly, and T1 (diesel) takes the first position when the weight of C7 is approximately 0.15. In this case, T2 (CNG) also increases rapidly, taking the first position when the weight of C7 reaches about 0.38.

**Fig. 4:**
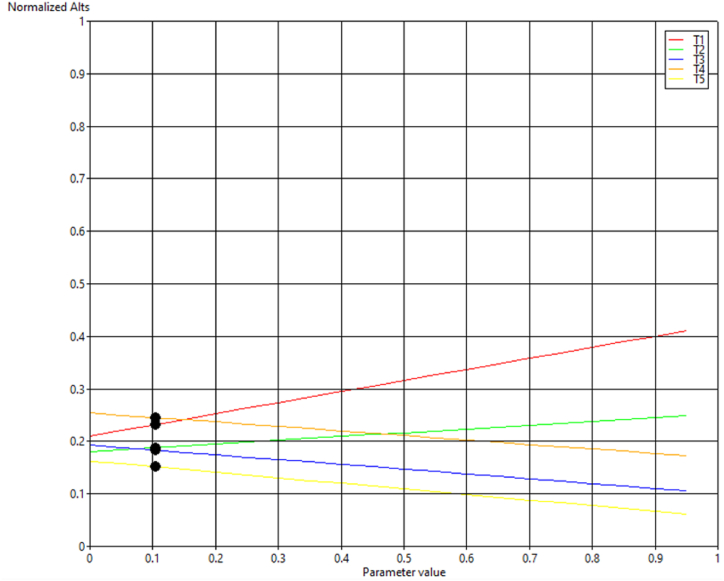
Sensitivity analysis of criteria C1.

Fig. 4 shows the sensitivity analysis of the first criteria (C1, vehicle cost). The rest of the sensitivity analysis figures have been included in the supplementary material.

The following section analyses and discusses the results obtained.

## 5 Discussion

The criteria chosen by the experts are aligned with those evaluated by other authors for the selection of waste management technologies in MCDM studies, according to the literature review carried out by Torkayesh [24]. That is why they can be grouped, following the dimensions of sustainability, in the categories defined by this author as economic (C1, C2), environmental (C4, C3) and social (C5). In addition, the author recommends other technical criteria specific to each study (C6, C7, C8, and C9 in this study).

The results show that the best-valued options are electric and diesel trucks, in this order and with a small margin of difference, followed by CNG and hybrid CNG-electric, and with hydrogen-powered trucks coming last.

The competitive advantage of alternative 1, electric truck (24.53%), is determined by its specific weight in the criteria of operating costs, atmospheric emissions, quieter operation, and social acceptance, with values between 34.1% and 40.2%. This result aligns with findings by other authors [12,87]. It should be noted that operating costs and atmospheric emissions represent 32.99% of the specific weight of the nine defined criteria. Despite the moderate and low ranking results presented by electric trucks in criteria such as configuration flexibility, ease of refueling (due to long recharge times), and lifetime (conditioned by the usually daily frequency of charging cycles for this type of vehicle), the results of the four criteria indicated above, combined with moderate specific weights in the criteria of investment costs and availability of spare parts, enable electric trucks to remain in first position.

The good results for diesel trucks, which are in second position and 1.31% behind electric vehicles (with a total 23.21%), are based precisely on their clear advantage in the rest of the criteria analyzed – with specific weights ranging between 26.8% and 50.0%. Diesel waste collection vehicles are highly developed, and their well-established position in the market (with a high level of competition) has produced machinery and spare parts at accessible prices. Almost any configuration is available, and the refueling methodology quickly recovers 100% of autonomy.

After these two alternatives, with very similar results, trucks powered by compressed natural gas (CNG) (18.77%) and hybrid CNG-electric (18.34%) are the third and fourth rankings (although based on different criteria). CNG-powered trucks have been progressively introduced in the Spanish market [88] and an increasingly wide range of configurations is available. At the same time, an increasing number of manufacturers have developed CNG vehicles and this has increased price competition – a criterion in which it obtains more specific weight than trucks that combine CNG with electric traction. The hybrid CNG-electric alternative, which uses fossil fuel to charge the batteries and electric motors for traction, shows better scores in the criteria related to environmental and social aspects and slightly better operating costs due to an optimization of the combustion engine operating regime. However, along with efficiency increments, hybrids come with other environmental impacts; for instance, they need more accessory materials per vehicle – including batteries [50,89].

It should be noted that compressed natural gas (CNG) (mainly methane) can also have a renewable origin, for example, from anaerobic digestion waste treatments. In this case, the assessments made by the experts would indeed have been different, as fuel generation would come from waste treatment processes that contribute to the circular economy. Liquefied biogas is a potentially important substitute for fossil fuels for heavy trucks [90] that has received little attention until now [91] and the results of a recent well-to-wheel assessment show that, compared to conventional fuels, in transport applications and for all vehicle classes (including heavy-duty vehicles), the use of compressed and liquefied renewable natural gas shows an 81% greenhouse gas emission reduction per kilometer traveled [92].

Finally, with a result of 15.15%, are hydrogen-powered trucks. This type of vehicle achieves the best results for atmospheric emissions [93] and relatively good specific weights for noise and social acceptance; however, its contribution to reducing greenhouse emissions depends on the energy mix used for its production [94]. Its low valuation in the rest of the criteria determines a lower valuation in global terms. It is a technology with little current implementation and an uncertain future [95] so both the offer and the variety of configurations is limited. Furthermore, this limited offer has reduced competition and so investment costs and options for spare parts are determined by a limited number of suppliers – meaning that this alternative is relegated to last place. Finally, high hydrogen production costs, a limited supply network, and inefficiencies in conversion to and from electricity [96], make this option the least attractive for the decision-maker.

Therefore, electric trucks are positioned as the best current alternative despite limitations in battery life, vehicle autonomy, and recharging times [97]. In agreement with other authors [47,48], electric vehicles can improve urban air quality, lessen climate change and reduce total energy usage. Diesel and compressed natural gas trucks are positioned as strong options [98] with superior evaluations in criteria such as investment costs, configuration versatility, ease of refueling, and lifetime – but fail to gain the top position due to lower social acceptance, noise, emissions [99], and operating costs (especially in the current context of rising fossil fuel prices). The hybrid combination of compressed natural gas and electric technologies is positioned close behind, as it is cleaner than pure combustion options [100]. However, it shows limitations due to the sum of the conditioning factors of compressed natural gas (supply points for refueling) and electric trucks (lifetime) and limits on possible configurations (higher weight due to CNG tanks and batteries). Finally, hydrogen trucks appear as the lowest-ranked alternative. Despite offering the lowest degree of atmospheric emissions [101] they are considered the least viable option due to their limited development, lack of competitiveness in the acquisition and aftersales market, and limited supply network.

If CST had not been used and only the current scenario (S1) had been considered, the ranking of alternatives would have been different (the diesel truck would be chosen with a considerable advantage over electric vehicles and compressed natural gas). This fact aligns with other SMCDM studies [25,38,102] and reveals the importance of considering the different feasible future scenarios when making decisions [24,39].

## 6 Conclusions

MCDM methods have often been used to deal with problems arising from MSW management, but they are usually focused on evaluating the best alternatives for waste treatment and disposal. However, from a sustainable and circular economy perspective, improving treatment techniques is insufficient, and waste collection and transport processes must also be reinforced. Municipalities are increasing MSW separation at source, and consequently, the collection routes grow in number (sometimes becoming door-to-door collections). Some MCDM studies have been made about choosing waste collection vehicles, but none that include the most recent green vehicles among the alternatives or consider feasible future scenarios.

This paper analyzes the five main vehicle motorizations in waste collection trucks (diesel, CNG, hybrid CNG-electric, electric, and hydrogen), considering sustainability criteria and using SBWM as a novel decision support model. SBWM is a multi-criteria method that combines two recently developed techniques: BWM and SMCDM. This method incorporates the probability of occurrence of feasible future scenarios in the decision-making process, which empowers decision-makers to express their judgments considering the uncertainty associated with decisions with long-term impact.

The results show that, despite their high price, electric trucks are already the best option for decision-makers, as they stand out in environmental criteria (emissions and noise) and social perception. However, the second ranked alternative, very close to the first, was diesel because of the ease of refueling, flexible configurations, and the fact that it offered the lowest investment costs.

SBWM offered reliable results and allowed dealing with uncertainty by considering different scenarios and enabling decision-makers to assign a likelihood of occurrence to possible future events. As sustainability criteria have been considered in evaluating alternatives, it can be concluded that SBWM has helped choose the best option to promote waste management sustainability by reducing the long-term impacts of mobility.

### 6.1 Limitations, recommendations, and future directions

This study has a few limitations that can be addressed in future works. One limitation is that only a few sustainability criteria were considered, especially for the social pillar. More balanced sustainability criteria, both qualitative and quantitative, should be considered in future works. Another limitation is that the criteria were assumed independent, which may not be the case. Future studies should address this issue using some methods that allow modelling the criteria interdependencies: ANP, DEMATEL, or interpretative structural modelling (ISM), to name a few. Moreover, to deal with uncertainty in criteria weights, decision-makers should be allowed to assign rating ranges or a value more an error instead of a single number when comparing criteria. Applying such a robust BWM in the future, we will add uncertainty to pairwise comparisons, making the decision-making process more realistic in current complex and ever-changing environments. Another limitation is that the proposed probabilities of occurrence assigned to the feasible futures may significantly affect the results obtained. Future studies should better inform decisions makers about future trends and relevant drivers of the most remarkable decision-making factors to allow more informed forecasting decisions about possible scenarios.

Regarding the case study, the findings may be somewhat limited by the application in a flat medium-sized city with a specific population density. The generalizability of the results will require collecting more experts’ opinions in different urban configurations and population distributions.

Finally, as future development, applying the stratification concept with other MCDM will allow comparing the results considering the consistency ratio. Comparing the results to other CST and MCDM combinations will improve the method's validation and test its applicability and usefulness.

## Author contribution statement

Héctor Moreno-Solaz: Concieved and designed the experiments; Performed the experiments; Analyzed and interpreted the data; Wrote the paper.

Miguel-Ángel Artacho-Ramírez: Performed the experiments; Analyzed and interpret the data; Wrote the paper.

Pablo Aragonés-Beltrán and Víctor-Andrés Cloquell-Ballester: Contributed reagents, materials, analysis tools or data.

## Data availability statement

Data will be made available on request.

## Declaration of competing interest

The authors declare no competing interests.
